# The Feasibility Study of Plasma-activated Water as a Physical Therapy to Induce Apoptosis in Melanoma Cancer Cells *In-vitro *

**DOI:** 10.22037/ijpr.2021.114493.14882

**Published:** 2021

**Authors:** Hamed Mahdikia, Babak Shokri, Keivan Majidzadeh-A

**Affiliations:** a *Laser and Plasma Research Institute, Shahid Beheshti University, Tehran, Iran. *; b *Tasnim Biotechnology Research Center (TBRC), AJA University of Medical Science, Tehran, Iran. *; c *Department of Applied Physics, Shahid Beheshti University, Tehran, Iran. *; d *Genetics Department, Breast Cancer Research Center (BCRC), Motamed Cancer Institute (MCI), ACECR, Tehran, Iran.*

**Keywords:** Plasma Gases, Reactive oxygen species (ROS), Reactive nitrogen species (RNS), Melanoma, Resazurin, Apoptosis

## Abstract

Low-temperature plasma (LTP) has demonstrated great potential in biomedicine, especially in cancer therapy *in-vivo* and *in-vitro*. Plasma activated water (PAW) as an indirect plasma therapy is a significant source of reactive oxygen and nitrogen species (RONS) which play an important role in apoptosis induction in cancer cells. In this study, Helium (He) plasma jet operating in 0.75 W and 20 kHz as dissipated power and frequency, respectively, is used as the cold plasma source. The electrical, thermal, and spectroscopic properties of (He) plasma jet and pH as well as the conductivity and temperature of PAW samples, are investigated. The concentration of hydrogen peroxide (H_2_O_2_), nitrite (NO_2_^-^) and nitrate (NO^-^_3_), which are produced in water as long-lived anticancer RONS, was measured 471.6, 7.9 and 93.5 μM, respectively after 6 min of plasma treatment. Alamar Blue and flow cytometry assays were employed to investigate the B_16_F_10 _cancer metabolic activity and apoptosis. These data support that cold atmospheric plasma (CAP) can produce a certain concentration of anti-cancer agents in water and induce apoptosis in melanoma cancer cells due to RONSs via activating the caspase 3 pathway.

## Introduction

B_16_F_10_ cancer cell line as known as melanoma cancer, is the most dangerous and aggressive cancer of the skin, which is spreading rapidly to other organs. Melanoma is metastatic cancer that can spread to the other part of the body and promote its survival (1). The global prevalence of cancers has been rapidly increasing during the last decades. Mortality and the insignificant response of melanoma to most standard therapies made it necessary to find alternatives to increase life expectancy (2). Chemotherapy, radiation therapy, immunotherapy, and photodynamic therapy are the common options for cancer treatment depending on the type of cancer (3). The most common and acceptable theory of anticancer effects of these therapies is based on oxidative stress and reactive oxygen species (ROS) accumulation which leads to cell death in cancer cells (4). The understanding of the importance of ROS in cell physiological functioning has changed over the last decades. ROSs were known as harmful and deleterious species (5, 6). Some evidence suggests that ROSs play a vital role in the intracellular function of cancer cells (7, 8). In fact, ROS plays a dual role in cancer due to its concentration in cells. ROS is required for cell proliferation, survival, cell signaling, and hemostasis. If ROS level increases in cells, they experience oxidative stress, so the excessive intracellular ROS can cause lipid, protein, DNA, and RNA damage and apoptosis (4, 9). Scientists are trying to find innovative methods to kill or stop the growth of cancer cells. During the past decades, non-thermal atmospheric plasma (NTAP) has shown great potential in cancer treatment. Plasma is an ionized gas with a temperature near room temperature. It consists of a set of charged and natural particles such as electrons, ions, ROSs, RNSs, free radicals, and other chemical factors, as well as some physical elements including electromagnetic waves, ultraviolet radiation, and thermal radiation (10). Findings indicate that RONSs generated by CAP induce anticancer effects with selective activity and other components seem to be negligible (11). Their efficacy was investigated in many different types of cancer cell lines including B_16_F_10 _(3, 12), SKOV-3 ovarian (13), pancreatic (14), LL/2 lung cancer (15), Cholangiocarcinoma (16), Osteosarcoma (17), and others as *in-vitro *and *in-vivo*. 

Previous studies have suggested medical plasma technology as a promising modality for melanoma treatment *in-vivo* and *in-vitro *(17, 18). Cold atmospheric plasma with helium and oxygen gas mixture demonstrated a significant modulation of metastasis-related markers like E-cadherin in human spheroids (20). 

Machala *et al.* reported that plasma gaseous products strongly depend on some physical parameters like the discharge regime, deposited power, and gas flow rate. They found that the streamer corona system leads dominantly to the formation of ozone and H_2_O_2_, while a more energetic transient spark is leading to nitrogen oxides and H_2_O_2 _(21). Ambient air is the most important and available source of RONS, which was initially generated by chemistry behind plasma–air interactions in the gas phase. The gaseous products determine the chemical properties of the PAW and the dominant aqueous RONS, so identification and kinetic investigation of these species will be valuable for future research in plasma–liquid interaction researches (21). The gaseous RONS are transported through the plasma-liquid interface and induce the formation of primary aqueous RONS like relatively long-lived hydrogen peroxide (H_2_O_2_), nitrite (NO^-^_2_), nitrate (NO^-^_3_) and ozone (O_3_) (22) and also short-lived, including the hydroxyl radical (OH), nitric oxide radical (NO), superoxide anion radical (O^-^_2_), hydroperoxyl radical (HOO^-^), nitric dioxide radical (N_2_O^-^), singlet oxygen (^1^O_2_), and ozone (O_3_) in the water interface and volume (23). These aqueous RONS then produce more cytotoxic products like peroxynitrites/peroxynitrous acid (ONOO^-^/ONOOH) and peroxynitrates/ peroxynitric acid (O_2_NOO /O_2_NOOH) through the chemical reactions, which have been shown to play a significant role in cancer therapy due to their triggering of cell death mechanisms (4, 9). Also, a mixture of different gases like H_2_, O_2_, and N_2 _to feeding gas could alter and modify the RONS concentrations (24). This mixture could produce OH, atomic O, and NO reactive species in the gas phase and a higher concentration of RONS such as H_2_O_2_, NO^-^_2_, NO^-^_3,_ and OH in the liquid phase (25). There are various different methods to detect and characterize free radicals and ROS in the gas and liquid phase like optical absorption spectroscopy (26), ion chromatography (IC) (27), liquid chromatography-mass spectrometry (LC-MS) (28), and UV–visible spectroscopy (UV–Vis) (23). Furthermore, the Electron Spin Resonance (ESR) technique has a great potential to detect and analyze ROSs produced by plasma in liquid, DMPO, and human tissue (29, 30).

Zhang *et al.* suggested that the He plasma jet caused physical damage in the central area of a cell culture well during the treatment but induced apoptosis in the peripheral region due to ROS production. They found that the effective anticancer area of the plasma jet directly depends on the gas flow rate when plasma is exposed directly to the media (31). According to Boehm and his colleague report, H_2_O_2_ concentrations can be correlated with increased cell toxicity and proliferation and can be used as an indicator of the potential efficacy of plasma-activated liquid (32). A synergy of H_2_O_2_ and NO_2_^- ^in cytotoxic effects on both normal and cancerous cells was reported by Girard and colleagues. They proposed this synergy as a basis for the cytotoxicity of plasma treatment with a He plasma jet device (33). Toxicity created by PAW is determined largely by cellular antioxidant status and the ability to detoxify H_2_O_2_ through catalase (32). Evidence demonstrated that reactive species in PAW, especially H_2_O_2_, can react with the biological component like proteins, lipids, nucleic acids and lead to mitochondrial dysfunction, cell cycle arrest, alteration in cell signaling, damaging cellular membrane, intracellular component enzyme, DNA, and trigger apoptosis (4, 9 and 34-37). Peroxynitrite acid, as known as the cytotoxic agent, is a secondary reactive species produced in plasma exposed liquids and has been reported to be responsible for biocidal effects in both microbial (38) and mammalian cells (39) systems. “Dehui XU” (40) made a systemic study on the safety of the immune-deficient nude mice that were treated by atmospheric plasma-activated water produced in a DBD reactor in ambient air. They injected 300 μl PAW into the mouse oral cavity for oral lavage 3 times and found that PAW reduct the tumor without any side effect on mouse vital organs.

Intracellular ROS cause some damage to important cellular components such as the cellular membrane, DNA, mitochondria, and endoplasmic reticulum and finally trigger apoptosis, necrosis, autophagy-associated cell death, or senescence (41). Specifically, activation of intracellular signaling leads to apoptosis due to intracellular ROS, creation of pores in the cell membrane, activation of the P53 protein, activation of the P21 CKS inhibitor, and stopping the cell cycle (42). Also, Cell apoptosis can be regulated with some proteins such as Bax and Bcl-2. Bax is a pro-apoptotic protein that promotes the cell death pathway through the promotion of mitochondria after exposure to cellular stress. In contrast, Bcl-2 as an antiapoptotic protein has an inhibitory effect on apoptosis via inhibition of Bax (43). The Bax/Bcl-2 ratio, which was evidence of the cell death progression by apoptosis, increased in the plasma-treated B16 tumor cells compared to the untreated control group (18). The expression level of apoptotic proteins like caspase 3 and caspase 8 and the relative Bax/Bcl-2 ratio in plasma-treated cancer cells in compared with the control group demonstrating the induction of the apoptosis mechanism (44). 

However, the synergistic mechanisms underlying these observations are still obscurant. This research aimed to investigate the trends of reactive species in the gas phase and the correlation between them with RONS produced in PAW. Optical emission spectroscopy (OES) was used to identify RONS in the gas phase. Long-lived reactive species include NO^-^_2_, NO^-^_3,_ and H_2_O_2_ concentrations, were measured using the colorimetric method. The mechanism and chemistry of RONS production in water were described. Also, the efficacy of the PAW on the treatment of melanoma cancer cells is demonstrated and discussed. Cell apoptosis using caspase 3 and metabolic activity of B_16_F_10_ murine melanoma cancer cells was done for the different treatment exposure times of PAW samples.

## Experimental

*Materials and methods*

*Plasma source and electrical characterization*

**Figure 1:**
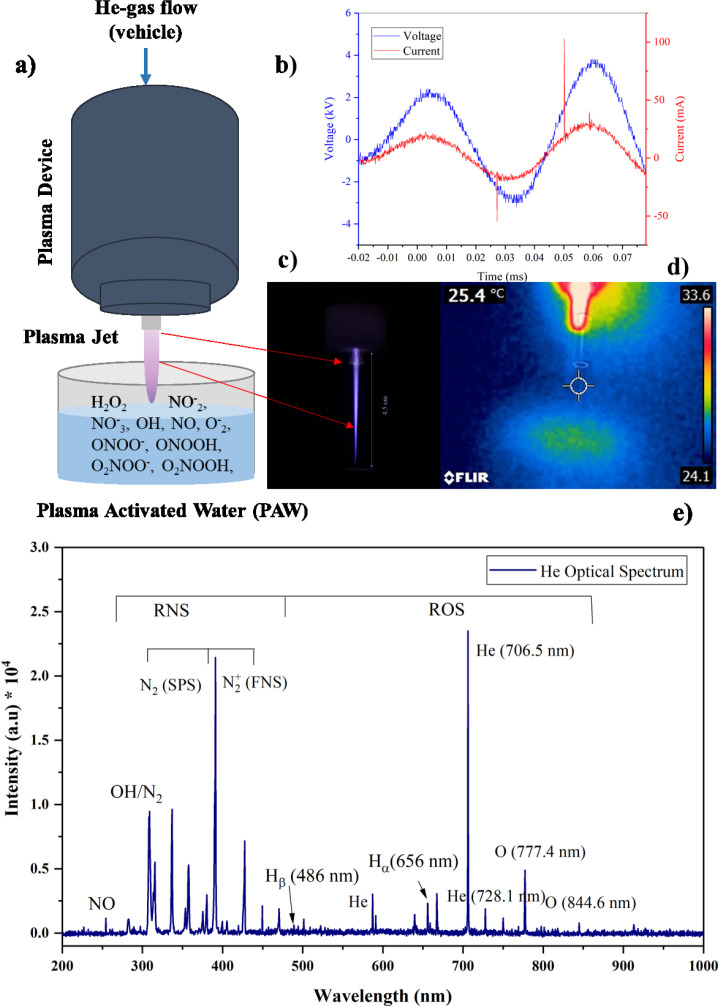
Physical characterization of plasma source during treatment of water: (a) schematic diagram of plasma source (b) representation of voltage (blue) and current (red) waveforms (c) He plasma jet used in this study (d) thermographic picture of plasma jet and (e) typical optical emission spectra of He plasma in air. All data was recorded at 4 kV and 20 kHz

In this study, the Plasma jet was operated with an AC power supply with 20 kHz pulse repetition frequency, and He (99.995% purity) was used as carrier gas with a flow rate of 4 standard liters per minute (slm). A schematic diagram of the plasma source was shown in Figure 1a. The gas discharge process occurs between the pipe and ring shape electrodes through a dielectric barrier. A homogeneous plasma is generated in the discharge gap of 3 mm and flows outwards through a nozzle as effluent. The waveforms of the discharge voltage and currents were recorded by HV probe (Tektronix, P6015A) and current probe (Tektronix, TCP202), respectively and measured using an oscilloscope (Tektronix, DPO 3012). The average power (P) was calculated from the current (I) and voltage (V) signals over a period (T) (45):

*Optical emission spectroscopy*

The plasma composition includes atomic and gaseous reactive species, was analyzed by real-time optical emission spectroscopy. Optical fiber and AVANTES spectrometer (Avaspec-3648-USB2 with a spectral resolution of 0.06 nm, grating with 300 lines/mm and 10 μm as entrance slit in the wavelength range from 200-1100 nm) were used to collect the emission spectrum of plasma. The optical probe was mounted 1 cm away in the perpendicular direction of the plasma jet and moved away in the same interspace from the plasma nozzle (Z = 0 mm) to the end of the plasma jet (Z = 45 mm), which guarantees a clear spectrum when detecting the emission spectrum. Data were analyzed and quantified by Avasoft 8.10.0 software (12). 

*Plasma activated water*

To prepare PAW samples, 750 μL of distilled water was treated for 2, 4, and 6 min with a He plasma jet 3 times. The distance between the surface of the treated liquid and the nozzle was set at 40 mm due optimum distance obtained from the OES result. The temperature of non-treated and treated samples was evaluated using a thermometer and, a non-contact thermographic camera (FLIR E4 camera 80 × 60 pixels) was employed to investigate possible thermal damage caused by plasma (19) The conductivity and the pH of PAW samples were measured using an electrical conductivity meter and pH-meter (TES-1381, Taiwan), respectively (45).

*RONS detection*

A colorimetric assay with titanium oxysulfate (TiOSO_4_) was performed to measure the H_2_O_2_ concentration based on the reaction of H_2_O_2_ with the titanium (IV) ions under acidic conditions (33). Fifty microliter of TiOSO_4 _was added to the 100 μL of samples and incubated for 15 min. Then, the UV absorption spectra of the yellow color of the produced pertitanic acid (H_2_TiO_4_) at 407 nm were obtained with a UV-1800 UV/Vis spectrophotometer. H_2_O_2 _concentration was calculated using a standard curve for each plate. Sodium azide (NaN_3_, 60 mM) was added to the samples before mixing with the titanium oxysulfate reagent to avoid the possible H_2_O_2_ decomposition by NO_2_^- ^under acidic conditions (33). Sodium azide immediately reduces NO_2_^-^ into molecular nitrogen and preserves the H_2_O_2_ concentration intact. Nitrogen included reactive species like NO_2_^-^and NO^-^_3 _were detected and concentrated using a colorimetric assay (ROCHE, Basel, Switzerland) with the same methods at 540 or 570 nm. The colorimetric reagent (diazo dye) was produced from the reaction of sulfanilamide with NO_2_^-^, and N-(1-naphthyl)-ethylenediamine (NED) according to the Griess assay’s mechanism. 

*Cell culture*

The murine metastatic melanoma B_16_F_10_ cancer cells (Pasteur institute, Iran) were cultured in a complete medium containing Dulbecco’s modified Eagles medium, DMEM high glucose (Gibco Co, USA), 10% fetal bovine serum (FBS) (Gibco Co, USA), 2% L-Glutamine, 1% penicillin and streptomycin solution (Sigma- Aldrich, USA) as antibiotics. When cells reached about 80% confluence, they were sub-cultured to ensure proper growth and health. 

*Metabolic activity and cell toxicity in culture model*

Alamar blue, also known as resazurin, is a non-toxic assay that was developed to investigate cell toxicity by transforming non-fluorescent blue dye into a highly fluorescent output. Viable cells can transfer resazurin to its reduced form by mitochondrial enzymes. Resorufin can be quantified using a fluorescence plate reader. In order to assess metabolic activity, 10000 cells were seeded onto 96-well plates within 1 mL complete DMEM media and cultured for 24 h without a media change. The untreated group was considered as a control group. In order to treat the cell with different PAWs (0 min, 2 min, 4 min, and 6 min), 100 μL of the medium was replaced with PAW and cells subsequently were transferred to the incubator for 24h. Reassuring was added at a final concentration of 100 μM and incubated for 2 h. Fluorescents signals were detected by a multimode plate reader at 

(46).

*Flow cytometry*

Caspase 3 antibody is generally considered as one of the last steps of cell death and an indicator of apoptosis. In this case, 50000 cells were seeded in 24- well plates overnight. The next day, cells were treated with PAW (0 min, 2 min, 4 min, and 6 min) and incubated for 24 h. After treatment, the cells were detached with trypsin enzyme, washed twice with Phosphate-Buffered Saline (PBS), centrifuged at 2000 revolutions per minute (RPM) for 5 min, and processed for caspase activity. Cell suspensions were incubated on ice for 30 min with caspase 3 (Thermo Scientific). In the following, cells were labeled with binding buffer. Flow cytometry analysis was done by BD- FACSCalibur flow cytometer (47).

*Statistical analysis*

The statistical analysis was done using GraphPad Prism 8 (GraphPad Software, USA) and results were compared using one-way analysis of variance (ANOVA). The differences of parameters between indexes were considered to be significant at a *P*-value of < 0.05.

## Results and Discussion

*Plasma characterization*

According to the recorded waveforms demonstrated in Figure 4b, the applied operating current and peak to peak voltage were measured at 50 mA and 6 kV, respectively. The pulse duration for both waveforms was about 45 μs, and the plasma jet device was working at 0.75 W power. This power is not too much high to produce a high-temperature plasma. The homogeneous plasma effluent with around 45 mm length at 4 slm gas flow rate is demonstrated in Figure 1c. Its temperature under this operating condition is about 25.4 ^o^C which confirms that it is cool. Figure 1e represents a typical He plasma emission spectrum which is recorded at distance Z = 3 cm from the nozzle. The interaction of plasma discharges with ambient air leads to the formation of a high concentration of gaseous RONS. According to Figure 1e, He as the carrier gas identified with a specific wavelength at 706 nm and atomic oxygen (^1^O) emitted peaks are shown in 777.4 and 844.6 nm, respectively. There are two hydrogen spectral lines related to H_α_ and H_β_ at wavelengths of 656 nm and 486 nm due to the humidity of ambient air in the spectrum. Besides, the OH and NO molecules are represented with peaks at 309 and 258 nm, respectively. Also, as reported by M. Simek *et al.* in the case of a plasma discharge in environmental air, vibrational excited N_2_ molecules emit violet light at wavelengths under 400 nm (second positive system, C^3^Π_u_→B^3^Π_g_) and above 400 nm (first negative system N_2_^+^) (48). A summary of plasma characterizations is listed in Table 1.

*pH, conductivity, and temperature of PAW*

**Figure 2:**
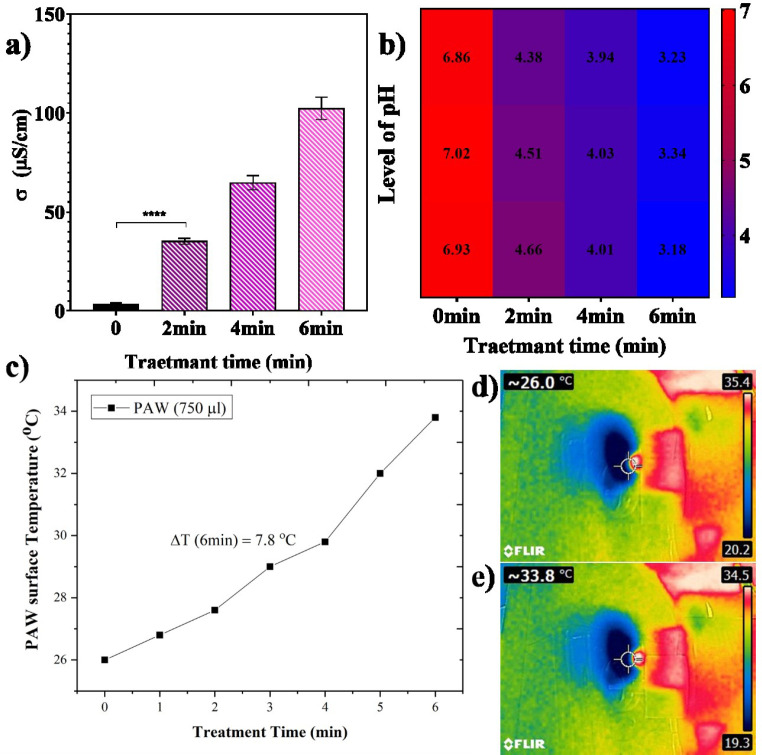
Physicochemical properties of plasma-activated water (PAW) after 6 min of plasma exposure at 0.75W. (a) conductivity (mean ± SD (n = 3)), (b) pH heat map and (c) temperature as a function of exposure time. and heat map of PAW (d) before and (e) after 6 min plasma exposure

The main reason for the acidification of water and its electrochemical properties after treating with cold plasma jet is mostly due to the production of H^+^ ions in PAW. The conductivity and pH of PAW are reported in Figure 2 as functions of exposure time with plasma jet. As shown in Figure 2a, conductivity increased from 3.16 μS/cm to 102.36 μS/cm due to electron and anion concentration produced in PAW and, the pH behaved the same but in the opposite direction after plasma treatment. In the PAW samples, the concentration of H^+^ ion is higher in competition with OH radicals. In this case, the pH level decreased to 3.25 after 6 min of plasma treatment (Figure 2b). It means that an increase in water conductivity is directly correlated with decreasing the pH level. The acidifying solutions with low pH values are crucial for cell toxicity effects (49). In addition, after 6 min of plasma exposure, the temperature increased up to 33.8 ^o^C that is lower than the survival temperature of 37^ o^C of cells under incubation conditions. It means that differences in the water temperature before and after plasma treatment do not affect the cell’s viability. Figures 2c-2e present temperature variation versus PAW exposure time and PAW temperature before and after plasma exposure. This result proves that plasma exposure produces an acidified medium at room temperature that is enhanced to induce toxicity in cancer cells.

*RONS* *concentration measurement*

**Figure 3:**
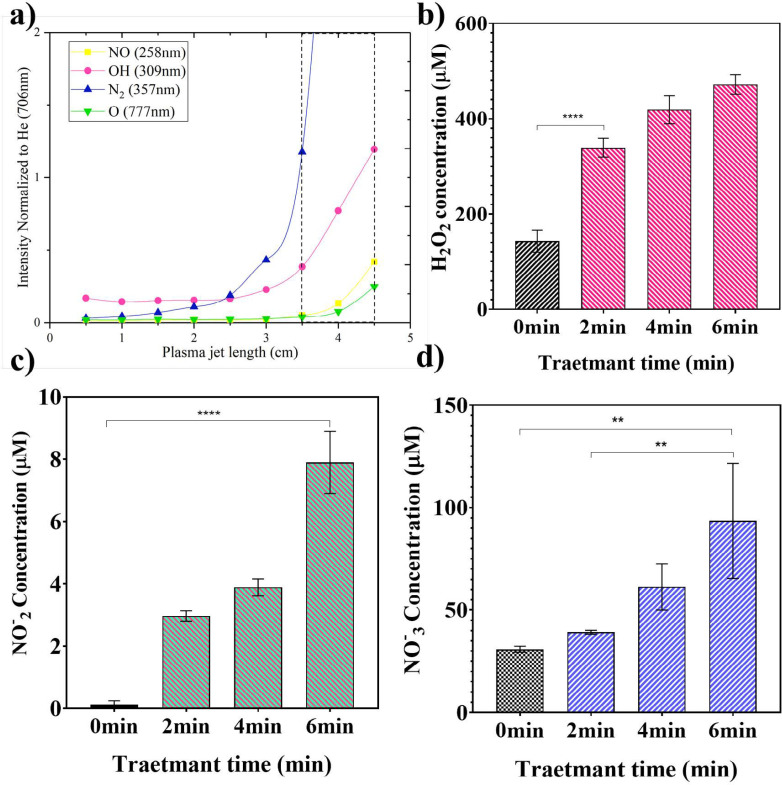
**Plasma treatment leads to the formation of RONS in the gas phase and liquid. (a) The trend of reactive oxygen and nitrogen species (RONS) intensities normalized to the He peak line at (706 nm) along with the plasma effluent. (b) H**_2_ **O**_2_ **, (c) NO** ^-^_2,_ **and (d) NO**_3_^-^ **, concentrations as a function of treatment time. Data are presented as mean ± SD (n = 3).**

Figure 3a presents a semi quantify trend of some RONS generated in the gas phase using OES. In this plot, the ratio of optical intensities related to the NO (258 nm), OH (309 nm), N_2_ (357 nm), and atomic oxygen O (777 nm) lines are normalized to the He line intensity at (706 nm) in vary interspace from 0.5 to 4.5 cm along with the effluent in the same applied power and gas flow rate. The results show that the normalized intensities of gaseous product generated in the plasma effluent tail at 3 cm from the nozzle are higher than in the closer distance to the nozzle and the interspace between 35 mm to 45 mm of effluent was an optimized source of RONS, so that the plasma source was set up at 40 mm above the water surface. As shown in Figure 3, according to our measurement, untreated water contains 0.1, 30.88, and 143 μM of NO_2_^-^, NO^-^_3_, and H_2_O_2_ RONSs, respectively. Therefore, a time-dependent irradiation increase of H_2_O_2_ as a product of OH (Figure 3b), NO_2_^-^ and NO_3_^-^ as products of nitric oxide (Figure 3c), and O (Figure 3d) was observed in the plasma-irradiated water. 

The concentration of generated RONSs increases with increasing the treatment time but not with the linear behavior. In the H_2_O_2_ case, the concentration reached to more than 2-fold of the initial value (340 μM) after 2 min of plasma treatment and, following, its molarity reaches to 418.5 and 471.6 μM for 4 min and 6 min treatment, respectively. The concentration of produced H_2_O_2_ is related to some parameters like surface to volume ratio of treated water, well diameter, treatment time, post-treatment storage time, and applied voltage (32). Machala *et al.* reported that the lower power leads to produce O_3_, H_2_O_2,_ and NO_3_^-^, while in the higher power, NO^-^_2_ generation is dominant (21). Findings indicate that in 0.75W power, NO^-^_2 _concentration is lower than other species. NO_2_^- ^was not produced in high concentration in comparison with H_2_O_2 _and NO^-^_3._ For nitrogen-containng RONS, molarities rise about 2-fold in a 6min case while their value is comparable for 2 min and 4 min plasma treatment. The final concentrations of NO_2_^- ^and NO^-^_3 _after 6min plasma exposure were measured 7.9 μM and 93.5 μM, respectively show that in the same condition, NO^-^_3 _concentration is higher than NO_2_^-^.

*Metabolic activity and morphology of cancer cells*

**Figure 4.**
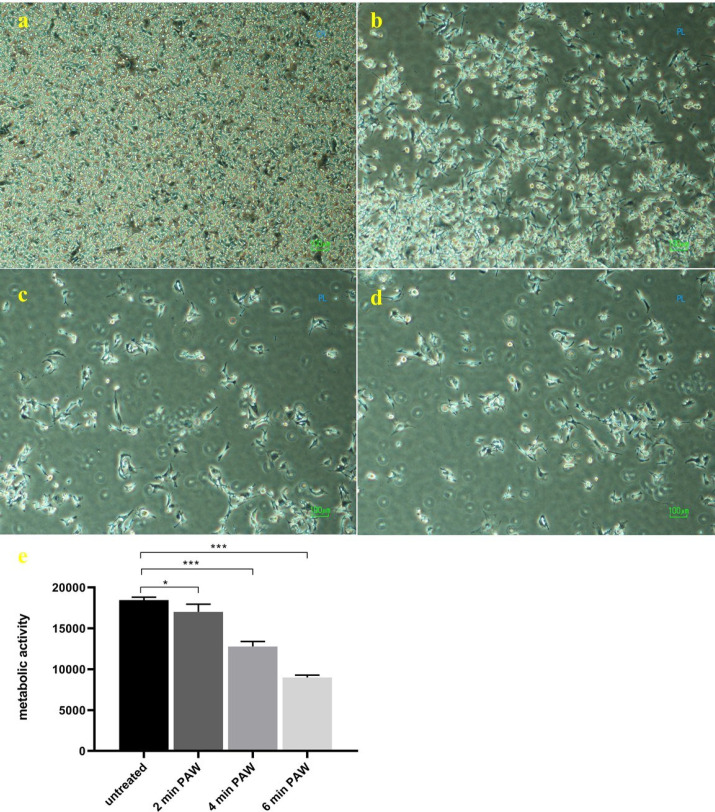
**Morphology and metabolic activity of B**_16_ **F**_10_ **cancer cells after incubated with PAW. Microscopic image of shape and concentration of cells after treated with (a) 0 min (b) 2 min, (c) 4 min, and (d) 6 min of plasma-activated waters after 24 h incubation. (e) metabolic activity of melanoma cancer cells after 24 h. Data are presented as mean ± SD (n = 3). Statistical analysis was performed using one-way analysis of variances with** ***p*** < 0.05 (*) and ***p*** < 0.001 (***); ns = non-significant

PAW in different exposure times was used for melanoma cancer cells treatment *in-vitro *(Figure 4). The PAW led to a significant increase in B_16_F_10_ cell death (Figures 4a-4d), and a significant decrease in the cell’s metabolic activity was observed at 24h post-treatment (Figure 4e). There is an acceptable conformity between metabolic activity results and morphological change in melanoma cancer cells. Cell death has been shown in optical microscopy as changes in cell morphology. It is characterized by a sequence of morphological changes like cell shrinkage, fragmentation of cells to small ones, membrane-bound, and enclosed by the surrounding cells like a bubble (50). According to Figure 4a the concentration and morphology of B_16_F_10 _cells in the control group is approximately fully dense and adhered to the plate bottom, while in PAW treatment groups, especially in 4 min and 6min PAW, the morphology and shape of cells are relatively smaller and more rounded. B_16_F_10 _cells indicated decreasing in metabolic activity after the incubation related to the NO^-^_2_ and H_2_O_2_ concentration in PAW. Thus the induction of apoptosis in cancer cells corresponds to the RONS concentration that is directly proportional to the plasma exposure time in PAW. Kurake *et al. *proved that despite H_2_O_2_, NO^-^_2_ has no significant anticancer properties lonely even in high concentrations up to 200 mM (21). In addition, the supplementation of cell cultures with NO_2_^-^ at low concentrations did not show cytotoxic effects on the tested cell line (32) while Peroxynitrous acid can be generated through the reaction of H_2_O_2 _and NO_2_^- ^at acidic pH and maybe the oxidative species, which induce a cytotoxic effect in cancer cells.

*Cell apoptosis*

**Figure 5:**
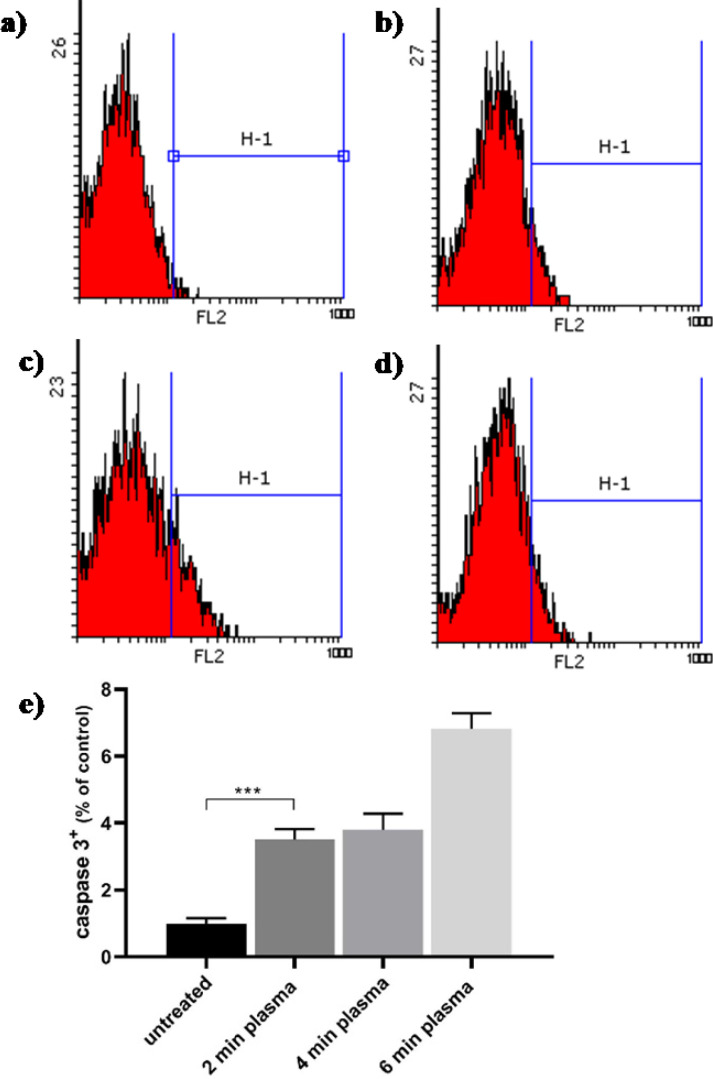
**Apoptosis of** **B**_16_ **F**_10_ **cancer cells treated by PAW. Caspase 3 histogram from flow cytometry of** **B**_16_ **F**_10_ **cancer cells** **after treated with (a)** **0 min (b) 2 min, (c) 4 min, and (d) 6 min of PAW** **. (e) quantifying the caspase 3** ^+^ **of melanoma cancer cells after 24 h. Data are normalized to the control group and presented as mean ± SD (n = 3). Statistical analysis was performed using one-way analysis of variances with** ***p*** < 0.05 (*) and ***p*** < 0.001 (***); ns = non-significant

One of the most relevant mechanisms of an anticancer approach is the induction of programmed cell death (apoptosis). Apoptosis consists of two pathways: The intrinsic pathway and the extrinsic pathway. Mitochondrial leakage of cytochrome c activates caspase 3 and initiates the intrinsic pathway. On the contrary, the extrinsic pathway involves the activation of membrane receptors that subsequently activate the caspase 8. To identify the death mode of PAW treatment in melanoma cancer cells, flow cytometry was subsequently employed. In B_16_F_10_ cells (Figure 5), PAW led to a significant decline in the percentage of cells negative for active caspases 3 (Figure 5e) being a marker of apoptosis. The expression of caspase 3 in the control group without adding the PAW has been demonstrated in Figure 5a and is about 2.03% (equivalent to normalized 1%). The percentage of caspase 3 experssion in B_16_F_10 _cells treated with different exposure times of PAW is normalized to the control group and is determined by 3.50%, 3.80%, and 6.82% for 2 min, 4 min, and 6 min PAW, respectively. The result suggested that increasing the exposure time can trigger cell injury due to PAW treatment and thus leading to apoptosis-associated caspase 3 activation. The initial level of ROS in cancer cells is higher than that of normal cells due to the high metabolism of cancer cells. Therefore, the ROS in cancer cells passes the threshold much easier than in the normal cells after exerting additional ROS stress using PAW. As a result, cancer cells experience stronger apoptosis than normal cells after plasma treatment (51). CAP within the production of RONSs and increasing a certain dosage of intracellular ROS can trigger a complex sequence of biological responses in tissues and cells (4). It annihilates cancer cells while inducing the lowest damage to normal cells. CAP leads to increases in both extra and intracellular RONS (50). It is confirmed that the presence of RONS and its product *in-vivo* and *in-vitro* induces oxidative and nitrosative stress and leading to apoptotic and necrotic death depending on the dosage (24). Recently a new model has been suggested which is based on aquaporins (AQPs) that is the only confirmed H_2_O_2_ channel in the cytoplasmic membrane (52). Biologists have confirmed that most cancer tissues tend to express more AQPs in the cytoplasmic membrane than in normal homologous tissues (53). After plasma treatment, the H_2_O_2 _produced by cold plasma spreads significantly faster in cancer cells than that in normal homologous cells (52). Multiple studies try to explain the reason for cancer cell’s sensitivity to CAP. They suggest that it is due to faster proliferation (54), changing lipid composition of the cell plasma membrane (55), lower concentration of cholesterol (56), weakened antioxidant, and higher ROS concentration in cancer cells rather than normal cells (57). Our results are in line with reports on other tumor cell lines; for instance, their efficacy was investigated in many different types of cancer cell lines, including B_16_F_10 _(3, 12) SKOV-3 ovarian (13), pancreatic (14), LL/2 lung cancer (15), Cholangiocarcinoma (16), Osteosarcoma (17), and others as *in-vitro* and *in-vivo*.

**Table 1:** A short summery of characterized physical parameters

**Parameter**	**Value**
Plasma jet temperature	~ room temperature
Electron temperature	0.55 eV
Power	0.75 W
Voltage and Current profile	sinusoidal and periodic
Current magnitude	~ 50 mA
Peak to peak voltage	6 kV

## Conclusion

Physico-chemical properties of plasma-activated water (PAW) induced cold atmospheric pressure plasma (CAP) jet with He gas were investigated. In this regard, the concentration of reactive oxygen and nitrogen species (RONS) generated in plasma-activated water was quantified following different plasma exposure times. The concentration of H_2_O_2_, NO^-^_2,_ and NO_3_^-^, in the PAW samples with a CAP jet at 0.75 W for the maximum exposure time of 6 min was measured 471.6 μM, 7.9 μM and 93.5 μM, respectively. It is demonstrated that, in the same exposure time, in comparison with NO_2_^- ^and NO^-^_3_, a higher concentration of H_2_O_2_ was produced. PAW decreases the metabolic activity of B_16_F_10 _cells with an increase in caspase 3 expression as an apoptosis marker after 24 h incubation. Conclusively, CAP productions such as OH and NO radicals and their secondary product can play an important role in indirect cancer therapy. Also, in synergy with other conventional methods, indirect-CAP could introduce as an agent to sensitize the tumor cells to chemo and radiotherapy. 

## Declaration of interest statement

The authors declare that they have no known competing financial interests or personal relationships that could have appeared to influence the work reported in this paper.

## Originality disclosure

The data presented in this manuscript are original and have not been published elsewhere.
